# Research Progress on Laser Powder Bed Fusion Additive Manufacturing of Zinc Alloys

**DOI:** 10.3390/ma17174309

**Published:** 2024-08-30

**Authors:** Fuxiang Meng, Yulei Du

**Affiliations:** School of Mechanical Engineering, Nanjing University of Science and Technology, Nanjing 210094, China

**Keywords:** zinc alloy, laser powder bed melting, formation quality, mechanical property, biodegradation

## Abstract

Zinc, along with magnesium and iron, is considered one of the most promising biodegradable metals. Compared with magnesium and iron, pure Zn exhibits poor mechanical properties, despite its mild biological corrosion behavior and beneficial biocompatibility. Laser powder bed fusion (LPBF), unlike traditional manufacturing techniques, has the capability to rapidly manufacture near-net-shape components. At present, although the combination of LPBF and Zn has made great progress, it is still in its infancy. Element loss and porosity are common processing problems for LPBF Zn, mainly due to evaporation during melting under a high-energy beam. The formation quality and properties of the final material are closely related to the alloy composition, design and processing. This work reviews the state of research and future perspective on LPBF zinc from comprehensive assessments such as powder characteristics, alloy composition, processing, formation quality, microstructure, and properties. The effects of powder characteristics, process parameters and evaporation on formation quality are introduced. The mechanical, corrosion, and biocompatibility properties of LPBF Zn and their test methodologies are introduced. The effects of microstructure on mechanical properties and corrosion properties are analyzed in detail. The practical medical application of Zn is introduced. Finally, current research status is summarized together with suggested directions for advancing knowledge about LPBF Zn.

## 1. Introduction

As the population ages and expands, bone diseases like osteonecrosis, osteoporotic fractures, and bone tumors are becoming increasingly prevalent [1]. Orthopedic surgery continues to face a considerable challenge in treating associated weight-bearing defects. Implants made from titanium alloy, tantalum, and polyether ether ketone are currently the most advanced clinical strategy. Nevertheless, permanent implants can cause chronic inflammation, reduced bone regeneration, and associated repair procedures [2,3]. Metal implants should degrade as bone regenerates and disappear when natural bone remodeling restoration function [4,5,6]. Biodegradable implants could facilitate bone healing and be gradually absorbed by the body after regenerating damaged bone tissue [7]. In the field of biomaterials, zinc has good prospects for future development, due to its ability to solve engineering problems such as excessive degradation of magnesium alloys, hydrogen release, slow degradation of ferroalloys, and cell toxicity related to Fe(OH)_3_ [8].

Zinc alloys are typically prepared through casting [9,10,11], which has the advantage of allowing composition control through melting, but controlling the element uniformity and complex geometry are difficult [12,13]. The process of casting involves heating metal components above their melting point, pouring liquid metal into the mold, and then solidifying the metal. Powder metallurgy (PM) combined with spark plasma sintering (SPS), osmosis casting and hot press sintering (HPS) are used to create porous zinc-based alloys. Capek et al. [14] used SPS to prepare porous Zn, and the average porosity of the sample was 20%. By using HPS sintering with NaCl template, Hou et al. [15] prepared porous biodegradable Zn scaffolds with the porosity of 75%. However, none of these methods can produce interconnected porous structures, especially when complex external structures are required, which is not conductive to cell penetration, blood vessel development, and nutrient diffusion [16]. Furthermore, it is difficult to control complex geometry and incorporate internal structures and curved channels into the sample using traditional manufacturing techniques, and organic solvents may be used during processing, which can impair cell viability and function [17,18].

**Figure 1:**
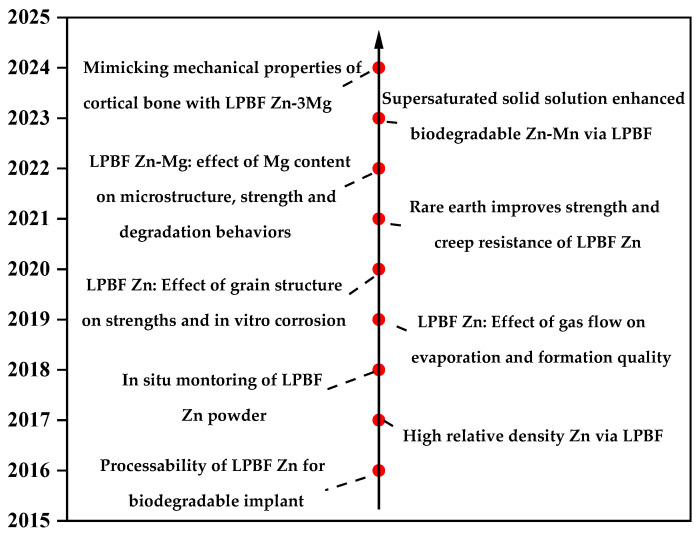
Development of Zn and Zn alloys prepared by LPBF [22,23,24,25,26,27,28,29,30].

Additive manufacturing (AM), also known as 3D printing, is a collection of production process that ASTM defines “a process of connecting materials from 3D model data to create objects” [19]. AM technology has the advantages of high manufacturing flexibility, high degree of automation, and no limitation of part structure, and is widely used in personalized small batch customization, aerospace, biomedicine and other fields. For 3D printing metal biomaterials, the two most popular techniques are LPBF (e.g., selective laser sintering (SLS) and selective laser melting (SLM)) and direct energy deposition [20,21]. Additive manufacturing of zinc alloys is still in the beginning stage, and researchers mainly use LPBF to prepare high-quality pure zinc, porous zinc scaffolds and various zinc alloys. There is no doubt that LPBF additive manufacturing of zinc alloys has attracted the attention of many researchers both at home and abroad. Figure 1 [22,23,24,25,26,27,28,29,30] shows the development of LPBF additive manufacturing of zinc alloys.

There have been many literature reviews on the biodegradable Zn [13,31,32,33,34,35]. Bowen et al. [32] reviewed the application of biodegradable Zn in biodegradable stenting. Venezuela et al. [13] focused on the relationship between the mechanical properties, biodegradability and biocompatibility of biodegradable Zn with alloying and manufacturing techniques. Recently, Zhou et al. [31] centred around the forming quality, microstructure characteristics and properties of bulk Zn alloys printed by LPBF. Yusop et al. [35] overviewed the effects of topological design and dynamic-flow corrosion on corrosion and in vitro biocompatibility of Zn-based scaffolds. The current review establishes on these works, introduces recent developments and provides some framework for future research. This paper highlights the revolutionary potential of biological systems in designing energy absorbers and provides a comprehensive review of various designs including as thin-walled structures, bio-inspired metamaterials, hybrid and hierarchical designs. It briefly reviews different optimization techniques for topology and crashworthiness optimization. Subsequent sections survey various characterization methodologies, such as mechanical testing and numerical simulations, and the parameters influencing the energy-absorbing behavior of LPBF-fabricated bio-inspired metamaterials, such as relatively density, base material, loading type, and strain rate effect. Additionally, it addresses the effect of conditions and manufacturing defects. A dedicated section discusses the effect of manufacturing process on numerical modeling and the incorporation of these defects through multi-scale modeling and data-driven modeling techniques. The concluding segment reviews application in the medical domains concerning energy absorption followed by a discussion on the challenges and future trends in this area.

## 2. Composition and Powder Design of LPBF Zn Alloy

### 2.1. Composition Design

Degradable metal bone implants are designed to meet three requirements: mechanical properties comparable to neighboring bone tissue, degradation properties consistent with tissue growth, and biocompatibility in line with clinical standards. Zn is considered as a prospective degradable orthopedic implant because its good biocompatibility and natural biodegradability [36].

Pure Zn exhibits extremely low yield strength, tensile strength, and plasticity (ε < 0.25%) [37,38]. Alloying refers to the process of adding impurities to improve the properties of metals. Mg has good biocompatibility and low elastic modulus (45GPa), and the Mg^2+^ produced can effectively promote the formation of new bone. Cu is a biocompatible element that is essential for human function and is also antibacterial [39]. It has been reported that the biocompatibility of Zn is not diminished by alloying with Cu [40,41]. Additionally, the incorporation of Cu can alleviate the pitting tendency of Zn, thereby achieving a moderate corrosion rate and a relatively uniform corrosion pattern [42]. Bakhshashi-rad et al. [43] reported that the addition of a small amount of Al element could not only maintain the positive biocompatibility of Zn, but also confer the antibacterial ability against Escherichia coli. Sotoudeh-Begha et al. [44] discovered that Mn has a beneficial impact on the biocompatibility of Zn.

The surface activity of rare earth elements and their large atomic radius allows them to interrupt grain growth and create stable compounds, which makes it easier to combine grain refinement with second phase strengthening [45]. Shuai et al. [46] added rare earth Nd element to LPBF Zn alloy, and found that the addition of Nd can improve fluidity of molten pool and reduce Zn evaporation. When Yang et al. [27] introduced the rare earth element Ce to the LPBF Zn alloy, they discovered that the alloy’s mechanical characteristics and creep resistance improved, and that it also had good cell compatibility and high antibacterial activity. 

In addition to alloying, the fabrication of metal matrix composites (MMCs) is an additional method for optimizing metal properties. SiC is a well-known biological ceramic with exceptional mechanical capabilities, strong biocompatibility, and favorable effects on osteoblasts among other reinforcing materials [47,48,49]. Gao et al. [50] innovatively introduced in-situ interface reaction into Zn-SiC metal matrix composites and obtained Zn-SiC bio-composite with tightly bonded interfaces by combining pre-oxidation and LPBF. The poor interfacial compatibility of carbon nanofiber (CNF), a potential reinforcement component for zinc implants, considerably lessens the enhancing impact. Yang et al. [51] used rare earth La element as a suitable layer for the contact between CNF and Zn matrix, and prepared La@CNF/Zn alloy with good interface bonding through LPBF, which has been proved to have excellent mechanical properties and anti-tumor activities. Table 1 summarizes the composition of Zn alloys printed by AM in recent years.

### 2.2. Powder Design

**Figure 2:**
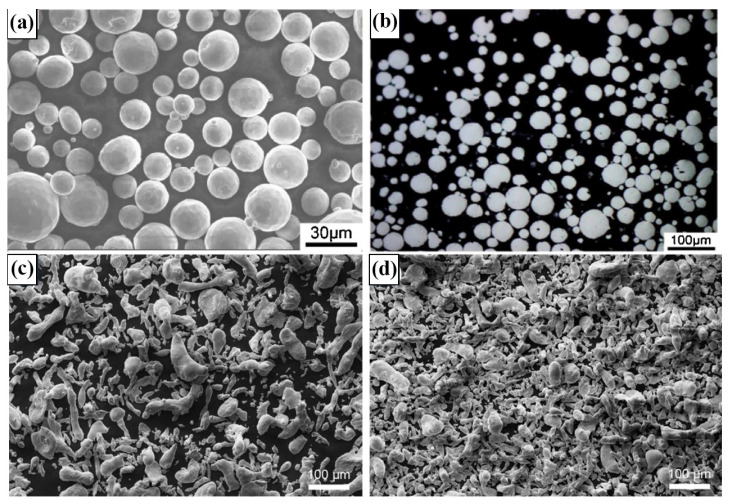
SEM image of pure zinc powder: (**a**) Nitrogen atomized pure zinc powder shape [52]; (**b**) Nitrogen atomized pure zinc powder cross section [52]; (**c**) Water atomized pure zinc coarse powder [23]; (**d**) Water atomized pure zinc fine powder [23]. (Reproduced with permission from Refs. [23,52], Elsevier).

The quality of metal powder significantly affects the final character of the formed component by AM. The ideal raw material for LPBF is spherical powder with good fluidity and spreading property. At present, the preparation methods of pure Zn spherical metal powder include vacuum induction melting gas atomization (VIGA), water atomization and plasma atomization. Gas atomization is a method of using high-speed inert gas to impact molten metal into tiny droplets, which are then condense into solid particles. Due to the effect of surface tension, the particles obtained by gas atomization are mostly spherical or nearly spherical. Wen et al. [52] prepared pure Zn powder by N_2_ atomization. The average particle size of the powder was 28.2 μm, as shown in Figure 2a [23,52].

The shape of the powder was uniform and most of it was spherical. The water atomization is a method of rapidly solidifying the metal into small particles through the impact of the water jet with the molten liquid of the metal. Demir et al. [23] adopted pure Zn powder formed by water atomization when preparing pure Zn by LPBF. The type of powder has irregular shape and high oxygen content. The coarse powder and fine powder separated by screen are shown in Figure 2c,d [23,52].

The common preparation methods of Zn alloy powder include mechanical alloying (MA) and pre-alloy casting with argon atomization. MA, which includes ball milling and vibration mixing, is a highly unbalanced manufacturing technique that artificially provides thermodynamic driving forces and mutual diffusion channels between different atoms [53,54,55]. Frequent welding, shattering, and rewelding of the combined powder during the MA process will significantly refine the particle size of the powder, but will give the powder an angular appearance and a rough surface. Shuai et al. [56] used MA to obtain heterogeneous Zn powder with an outer layer of nanoparticles and an inner layer of micron particles. Qin et al. [57] tried to prepare Zn-0.7Li powder by pre-alloy casting and argon atomization process for the first time, and the powder after atomization had good fluidity and uniform particle size distribution.

## 3. Additive Manufacturing of Zinc Alloys

### 3.1. LPBF Additive Manufacturing Technology

**Figure 3:**
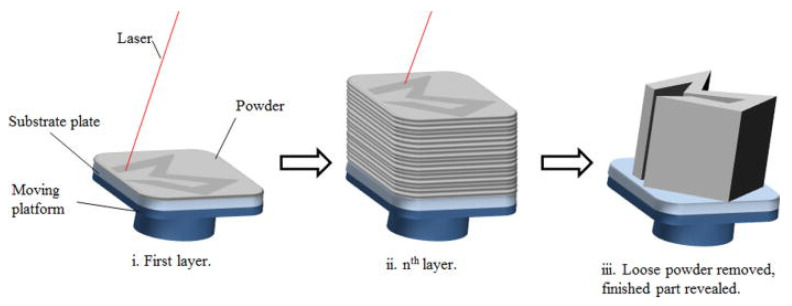
Concept of SLM process. (**i**) High-powder melts selective areas of the powder bed. (**ii**) Process is repeats for successive layers. (**iii**) Loose powder removed and finished part revealed. (Reproduced with permission from Ref. [58], AIP Publishing).

SLM is an LPBF process that selectively melts and fuses material layer by layer in accordance with computer aided design (CAD) data, as previously mentioned. The SLM process is a sequence of stages that spans from the preparation of CAD data to the removal of prefabricated parts from the building platform. In order to provide a support structure with overhang features and to produce slice data for laser scanning the layers, the STL file, prior to the CAD data being uploaded to the SLM machine for specimen printing. The construction process commences with the application of a fine layer of metal powder to the substrate of the molding chamber. When the powder is laid, the designated area is melted and fused using a laser in accordance with the processed data. Once the laser is finished, the building platform is lowered, and the next layer of powder is deposited on top. The laser then scans the new layer. This process is repeated in successive layers until the specimen is completely constructed. With the exception of data preparation and removal of the construct from the building platform, the entire process is automated. Figure 3 [58] illustrates the concept of the SLM process.

SLS produces parts with low strength and high porosity by binding particle materials through melting of binding agents or solid-state sintering. Consequently, it is frequently necessary to enhance SLS samples via post-processing (e.g., heat treatment and material infiltration), which significantly extends the manufacturing process. SLM, on the other hand, is capable of generating an entirely dense near net-shape component that typically requires no post-processing.

### 3.2. LPBF Zinc Alloy Formation Quality

The formation quality of LPBF parts is primarily determined by densification and surface roughness. Relative density represents densification, and arithmetical mean height of an area (Sa) is used to indicate surface roughness. A decent formation quality is typically indicated by a high relative density, and the opposite is true. Surface roughness considerably impacted the interaction between implants and cells. Due to particles that is adhered to the surface of AM components, AM parts cannot be used directly in medical applications [59]. Wen et al. [25] measured that the Sa on the side of the LPBF pure Zn cube was 9.15–10.79 μm, and the Sa decreased to 4.83 μm after sandblasting. This paper aims to investigate id post-processing of LPBF Zn scaffolds will effectively affect their degrading performance and biocompatibility. On this point, there is less literature research.

**Figure 4:**
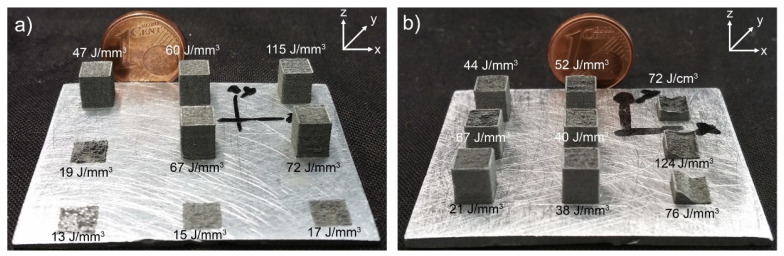
Appearance of pure Zn parts produced by LPBF: (**a**) missing parts due to low fluence and incomplete melting with coarse powder; (**b**) missing parts due to excessive fluence and explosive behavior with fine powder. (Reproduced with permission from Ref. [23], Elsevier).

The initial peer-reviewed paper on LPBF pure Zn was published by Montani et al. [22]. Its maximal relative density is only 88%, because the laser beam is attenuated by tiny particles in the vaporized smoke [60]. Particles in the evaporated smoke may land on the transmission mirror, blocking the movement of laser beam. The attenuation of laser beam leads to the collapse of the keyhole and an unstable process, resulting in a significant increase in porosity. Subsequently, Demir et al. [23] employed LPBF to fabricate pure Zn via auxiliary purging airflow. When volume energy density (E_v_) is in the range of 40–115 J/mm^3^, the cube components achieve a relative density of almost 98%, as depicted in the Figure 4 [23].

In addition, the study indicates that the formability of the powder with a large particle size surpasses that of the powder with a small particle size, since the fine powder is more responsive to variations in laser energy. Interestingly, the author also observed that the powder with finer particle size exhibited poor formation quality during the LPBF Zn alloy process.

The process parameters are closely related to the forming quality of AM metal, among which the most studied parameters are laser power (*P*), scanning rate (*V*), hatch spacing (*Hs*) and layer thickness (*Ds*), which collectively determine E_v_. The Equation (1) is as follows:

(1) $$E_{v}=\frac{P}{VHsDs}$$

**Figure 5:**
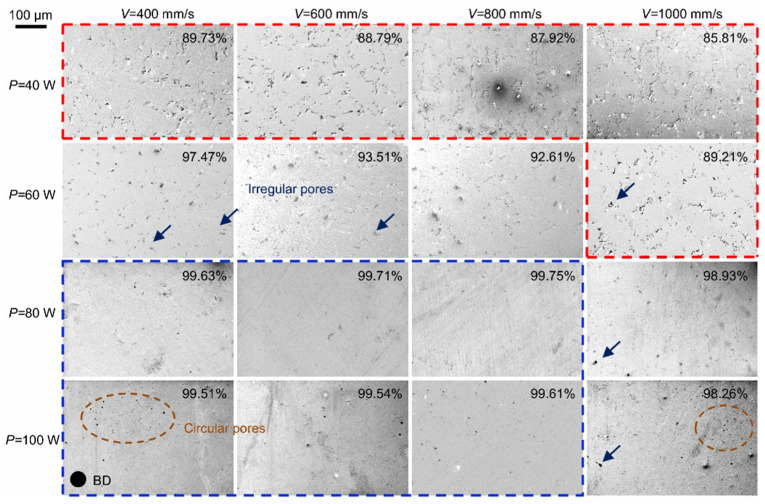
Cross-section morphology and relative density of pure zinc prepared by selective laser melting at different laser power and scanning speed. The scale bar is shown at the top left corner; the red-colored and blue-colored areas represent parts with a density below 90% and above 99.5%, respectively. The arrow refers to irregular pores. (Reproduced with permission from Ref. [62], Springer Nature).

In general, the influence of laser energy on forming quality is categorized into three zones with distinct E_v_ [61]. If the E_v_ is overly high, it will result in an excessive amount of evaporation. If the E_v_ is insufficient, the energy is inadequate to completely melt the entire powder. Yang et al. [62] conducted a comprehensive investigation on the influence of *P* and *V* on the forming quality of LPBF pure Zn. Figure 5 [62] displays the cross section of a polished Zn sample.

At a laser power of 40 W, regardless of the scanning speed, noticeable irregular pores are present on the cross section, suggesting that the E_v_ is inadequate. As the laser power increases, the irregularity of the pores decreases dramatically at an equivalent rat. When the power is increased to 100 W, circular pores that are randomly dispersed emerge on the surface of the cross section. At a scanning rate of 400 mm/s, a significant quantity of circular pores emerges in the cross section as a result of substantial Zn evaporation. Furthermore, When the laser power density (laser power divided by the area of laser spot) surpass the threshold of typically 10^6^ W/cm^2^, a narrow cavity is formed as a result of the of evaporation recoil. This defect is commonly known as a keyhole [63].

For the defects existing on the surface of the alloy, some researchers believe that it depends on the fluidity of the molten pool [64]. The fluidity of the molten pool is directly affected by the energy and scanning rate. Increased laser energy can improve the wettability of the molten pool, while a slower scanning can extend the interaction between laser and powder, resulting in a wider molten pool. The width of the molten pool usually ranges from 2 to 3 times the diameter of the spot. The depth of the molten pool is inversely proportional to the melting temperature of alloying elements [65,66]. This aligns with research conducted by Voshage et al. [28]. In the LPBF process, the molten pool depth of Zn (melting point 420 °C) and Zn-Mg (melting point 364 °C) is 114 μm and 200 μm, respectively. Notably, when the depth of the molten pool greatly exceeds the width, the formation of a keyhole is highly likely [67].

**Figure 6:**
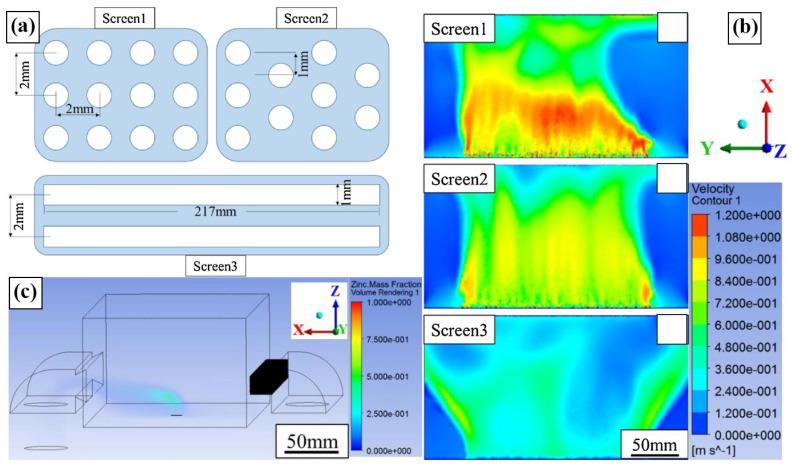
The structure of three kinds of circulating gas filters and their airflow rate distribution, and the optimization of zinc vapor mass distribution under the airflow: (**a**) different structures of blow-off screens; (**b**) velocity distribution with different types of blow-off screens; (**c**) mass fraction contours of Zn metal vapor under the optimized gas flow. (Reproduced with permission from Ref. [71], Laser Institute of America).

Apart from the effect of the above indicated process parameters on the forming quality, it is important to consider the factors (e.g., gas filtration structure, scanning strategy and protective gas flow rate) which affect Zn evaporation. Zn exhibits a severe tendency to evaporate, during prolonged laser melting, evaporation may be intensified, which could finally be connected to the high porosity resulting from burning Zn atoms [68,69]. Regarding the evaporation of specific components of the alloy, Klassen et al. [70] argue that it is directly correlated with the equilibrium vapor pressure of the component and inversely proportional to its molecular weight. Grasso et al. [24] applied infrared cameras to record the dynamics of smoke and splash while manufacturing Zn with LPBF. As the concentration of smoke from Zn evaporation rises, the porosity experiences a substantial increase. Chen et al. [71] utilized finite element software to simulate and analysis the effect of the circulating gas filter’s construction on the flow rate of protective gas. According to Figure 6 [71], the speed of first air flow is too high, the flow rate of the second gas is nearly constant, and the third air flow diverges after a certain distance from filter.

Mostaed et al. [60] found that Zn-0.5Mg printed by single point exposure (SPE) could inhibit the occurrence of excessive smoke and sparks during the LPBF process. There is a lack of literature to study the correlation between the speed of the protective and the evaporation and deposition of Zn. Due to the easy evaporation of Zn powder, the level of protective wind speed during the printing process is crucial to whether the Zn powder can be deposited on the substate plate.

Until now, the majority of research on densification has focused on bulk Zn, rather than Zn porous scaffolds. Previous Zn investigations using LPBF have shown that actual porosity of the printed scaffold differs significantly from the intended porosity. And these scaffolds are attached a lot of powder, which exhibits poor surface roughness. One factor contributing to the poor forming quality of the scaffold is the narrow scanning path, which prevents multiple scans by the laser. The laser spot size of the commonly used LPBF device is 80 μm, whereas the maximum attainable support is approximately 300–500 μm. The scanning strategy is crucial for the densification of porous scaffold, since it requires little trace of powder melting for each layer of the scaffold. Concentric scanning has been found to be more appropriate for producing fine structures compared to linear scanning. The reason for this is that the laser energy required for concentric scanning is lower than for linear scanning. Bagheri et al. [72] proposed the functional relationship between the deviation of the porous support column and building angle, and developed a compensating scanning strategy.

Table 2 [25,26,46,52,57,62,73,74,75,76,77,78,79,80] provides a concise overview of the process parameters and relative density of Zn alloy in recent years. Due to the different printing equipment, powder quality, and operators, for various components of Zn alloys, the E_v_ of 39.7–486.1 J/mm^3^ can obtain a high relative density.

### 3.3. Microstructure of LPBF Zinc Alloy

Microstructure plays an important role in the study of metal materials. Various factors such as process parameters, powder condition, and printing environment have an impact on the microstructure of the alloy, and the microstructure is closely related to the mechanical properties, degradation properties and biocompatibility. Researchers commonly use scanning electron microscope (SEM) to examine the surface morphology of untreated bulk samples. Following the polishing of the sample’s surface with sandpaper, the optical microscope (OM) was used to observe the quantity of pores and the shape of defects. The optimum surface of the sample was mechanically and electrolytically polished, and the texture and grain morphology were analyzed using an electron back-scattered diffraction (EBSD), and the microstructure was examined via transmission electron microscope (TEM).

**Figure 7:**
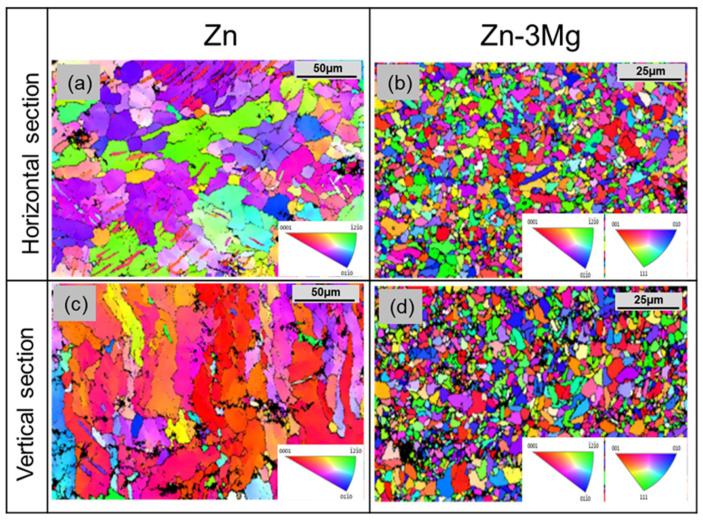
EBSD results of pure Zn and Zn-3Mg alloys: (**a**) surface of Zn; (**b**) surface of Zn-3Mg; (**c**) side of Zn; (**d**) side of Zn-3Mg [78]. (Reproduced under a CC-BY).

The temperature gradient (SR) and solidification rate (TR) are the primary factors that determine the grain morphology of LPBF Zn [80,81]. The grains tend to grow along the building direction with the largest temperature gradient, forming a columnar shape [82]. The LPBF pure Zn sample is a slender columnar crystal in the vertical direction, as illustrated in Figure 7c [78].

Figure 7a [78] illustrates that LPBF pure Zn exhibits large equiaxed grains in the horizontal direction. Nevertheless, such crystals are not found in Zn-3Mg because the generation of the Mg_2_Zn_11_ phase along the grain boundaries prevents the equiaxed grains from further developing into columnar grains. It is also evident that the addition of Mg element converts pure Zn with an average grain size of 21.1 μm into a Zn-3Mg alloy with an average grain size of 2.1 μm.

**Figure 8:**
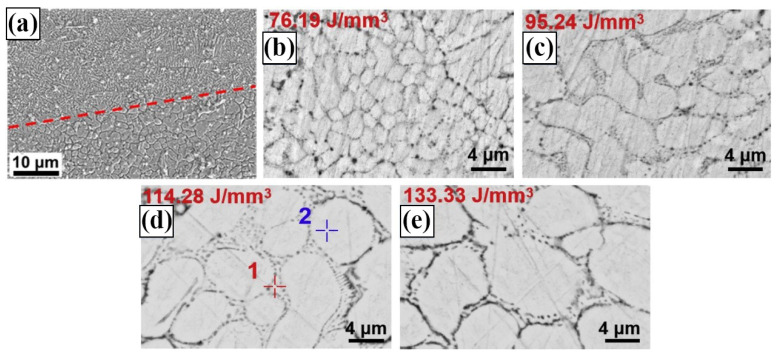
Microstructure of Zn alloy: (**a**) Zn-2WE43 [75]; Zn-2Al cross-section at (**b**) 76.19 J/mm^3^; (**c**) 95.24 J/mm^3^; (**d**) 114.28 J/mm^3^; (**e**) 133.33 J/mm^3^ [77]. (Reproduced with permission from Refs. [75,77], Elsevier).

The uneven distribution of powder particles will result in the microstructure exhibiting distinct alloy phases, as shown in Figure 8a [75,77].

The lower surface of Zn-2WE43 alloy consists of large and irregularly shaped α-Zn grains, while the upper surface is composed of dine α-Zn grains and Mg_2_Zn_11_ precipitates. This phenomenon is expected to have detrimental consequences on the mechanical characteristics, degradation properties, and biocompatibility of the alloy. The temperature dynamics during the LPBF process can affect the grain structure. Shuai et al. [77] found that Ev of the Zn-2Al alloy is 76.19 J/mm^3^, 95.24 J/mm^3^, 114.28 J/mm^3^, and 133.33 J/mm^3^, corresponding to grain sizes of 2.21 μm, 4.49 μm, 5.53 μm and 6.62 μm, respectively. At low Ev, the temperature of the molten pool is relatively low, resulting in a higher cooling rate of heat conduction. The quick advancement of the solid-liquid interface significantly inhibits the growth of grains, leading to the formation of relatively small grains. With the increase of E_v_, the heat accumulation in the molten pool is enhanced, which reduces the cooling rate. The extended cooling period facilitates the evaluation of the kinetics qualifications for grain growth, which leads to grain coarsening.

## 4. Properties of LPBF Zinc Alloy

### 4.1. Mechanical Properties

**Figure 9:**
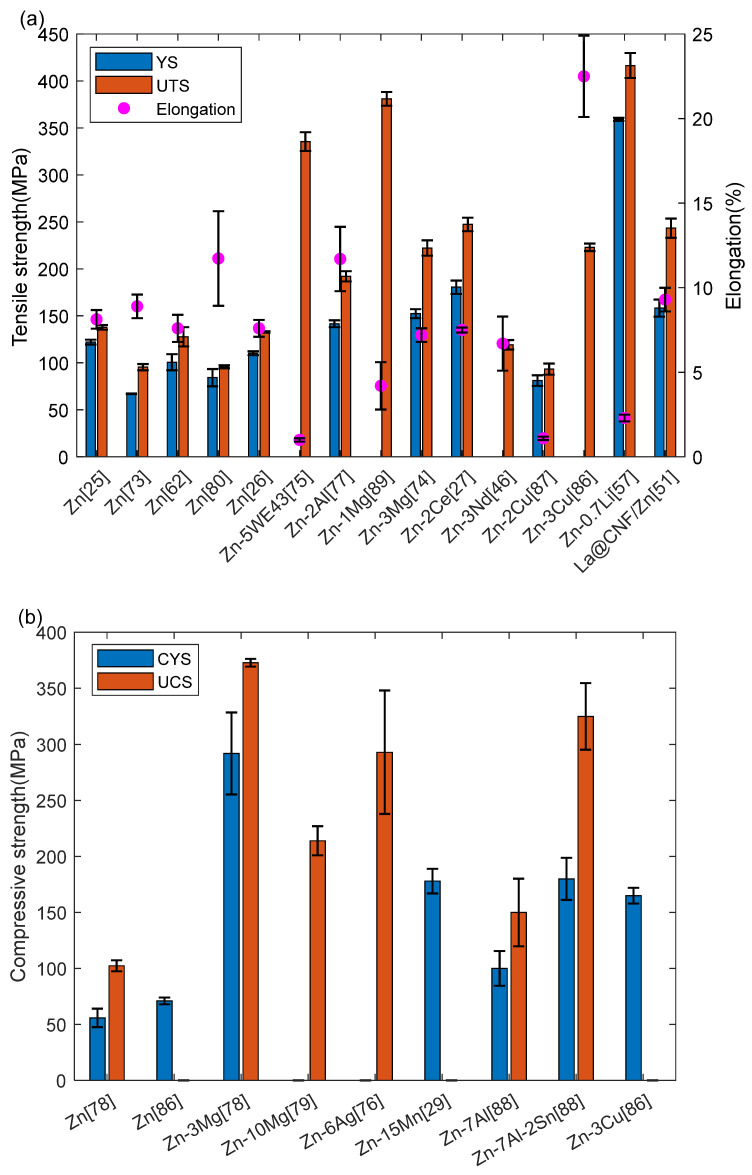

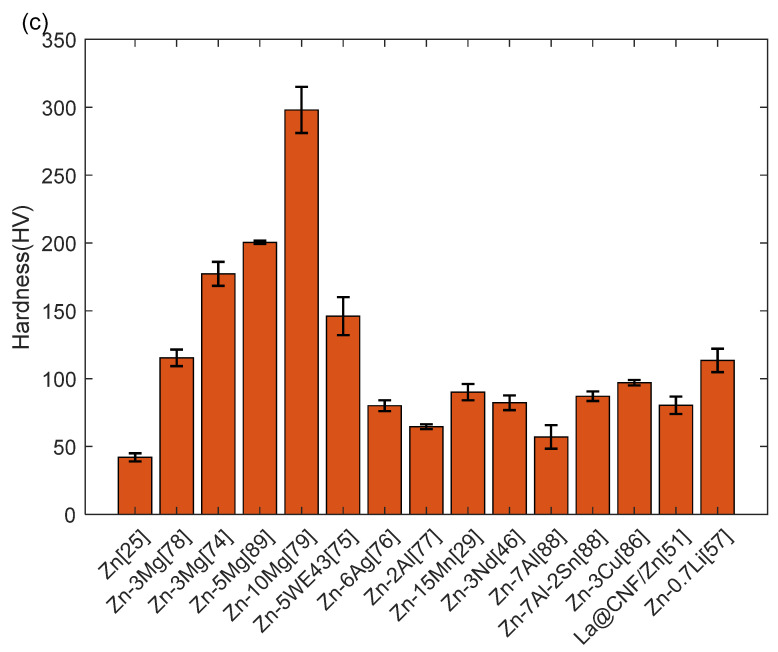
Mechanical performance of pure Zn and Zn alloys: (**a**) Tensile test [25,26,27,46,51,52,57,62,73,74,75,77,80,86,87,89]; (**b**) Compression test [29,76,78,79,86,88]; (**c**) Microhardness [25,29,46,51,57,74,75,76,77,78,79,86,88,89].

Among the biodegradable metals, Zn is not recognized for its attractive mechanical characteristics. Specifications for artificial bone implants: Ultimate Tensile Strength (UTS) ≥ 100–150 MPa, Compressive Yield Strength (CYS) ≥ 130–180 MPa, elongation ≥ 1–36% [83]. The stent typically consists of a narrow cylindrical tube with a diameter ranging from approximately 2.5 to 3.0 mm, and a thickness of support measuring between 70 and 175 μm [84,85]. This paper provides a summary of the mechanical properties of Zn alloys and its porous scaffolds, specifically focusing on the yield strength, ultimate tensile strength, elongation, compressive yield strength, ultimate compressive strength, and microhardness of bulk LPBF zinc alloys in recent years. The information is presented through Figure 9 [25,26,27,29,41,46,51,52,57,62,73,74,75,76,77,78,79,80,86,87,88,89].

The deficiencies in tensile strength, compressive strength and elasticity of LPBF Zn alloys can be improved by various approaches (e.g., alloying, adjustment of process parameters and post-processing). Nevertheless, Zn alloys have substantial anisotropy in their mechanical properties, which makes the joints of porous scaffolds easy to break. For example, Qin et al. [26] discovered that the YS and UTS of pure Zn in vertical samples were merely 69% and 68% of those in horizontal samples, respectively. Depending on the clinical requirements, the ideal orthopedic metal implant must offer mechanical reinforcement for 12 to 14 weeks [90].The anisotropy of Zn might lead to premature failure of the implant, which can be deadly and hinder its practical application.

Additionally, Zn alloys have challenges related to age hardening and strain rate sensitivity. Jin et al. [91] reported that the tensile strength of Zn-0.08Mg increased from 266 MPa to 498 MPa, while the plasticity dropped from 29.8% to 12.7% after 9 days of ageing at ambient temperature. After a period of 12 months, the alloy exhibits a tensile strength of 434 MPa and an elongation of 3.5%. This instability of mechanical properties over time is a concern, which greatly restricts the usability of biodegradable Zn alloys.

The ideal elastic modulus of bone scaffold should closely match that of normal bone, which ranges from 0.01 to 2 GPa [92]. An excessively high elastic modulus leads to stress shielding, which causes bone resorption and hinders bone repair [93]. Based to the Gibson-Ashby theory, there exists a negative correlation between the elastic modulus of porous scaffolds and their structural porosity [94]. Since scaffolds with different unit cell structures have different porosity, the elastic modulus of scaffolds can be adjusted by modifying the printing structure. The elastic modulus of Zn alloys varies throughout different studies due to differences in porosity, even when the alloy composition remains constant. Indeed, the elastic modulus is influenced by numerous additional factors. Table 3 [28,30,57,75,89,95,96] presents the compressive properties of Zn alloy porous scaffolds during the past few years.

### 4.2. Biodegradation 

When assessing implant metals, biodegradability and biocompatibility are the crucial factors. Both of these can be evaluated in vitro, which means outside the typical biological environment, or in vivo, which refers to testing in animals or humans. Electrochemical polarization testing (e.g., potentiodynamic polarization experiment) and immersion testing are the two most common techniques for in vitro biological corrosion testing. Potentiodynamic polarization experiment utilizes a three-electrode device comprising a working electrode (the metal under investigation), a reference electrode (e.g., a standard calomel electrode), and an opposing electrode (e.g., platinum). The test electrolyte is a fluid that replicates the physiological conditions, such as phosphate-buffered saline (PBS), equilibrium salt solutions (BSS) like Ringer’s (RBSS) and Hank’s, and simulated body fluid (SBF) [97]. Actual human plasma and entire blood are utilized in some degradation tests [98]. An electric potential is provided to the working electrode, and the output current is observed. The corrosion potential (E_corr_) and corrosion current density (i_corr_) are determined by scanning the potential within a certain range at a set scanning rate. Corrosion rate (CR) can be obtained according to Equation (2):

(2) $$CR=3.27\times 10^{3}\frac{i_{corr}EW}{\rho }$$

where i_corr_ (μA·cm^−2^) is the corrosion current density, EW (g·eq^−1^) is the equivalent weight, and ρ (g·cm^−3^) is the density of the metal. Another popular electrochemical test is electrochemical impedance spectroscopy (EIS) [99]. EIS uses alternating current (AC) polarization and direct current (DC) polarization in traditional potentiodynamic testing. EIS can obtain i_corr_ by polarization resistance, R_p_ and Stern-Geary relationship:

(3) $$i_{corr}=\frac{\beta_{a}\beta_{c}}{2.303R_{p}\left(\beta_{a}+\beta_{c}\right)}$$

where β_a_ and β_c_ are the anodic and cathodic Tafel slopes. This value of i_corr_ may be used to measure the corrosion rate, CR, according to Equation (2).

Immersion testing refers to the immersion of the test material in a corrosive environment for a specific duration, usually in a physiological liquid. The reported tests are typically conducted under static flow circumstances, and the test solution is then substituted with a fresh solution following a specific duration of soaking. Zn in the corrosion process causes an increase in the pH value of the solution [100], which subsequently impacts the corrosion rate of the measured metal. Once the designated duration of soaking has elapsed, the sample is extracted and the corrosion products are eliminated in order to measure their weight. Corrosion rate, CR (mm/y), can be calculated the Equation (4):

(4) $$CR=K\frac{W}{At\rho }$$

where K is a constant, W (g) is the mass loss of the specimen, A (cm^2^) is the surface area, t (h) is the time of exposure, and ρ (g·cm^−3^) is the density.

**Figure 10:**
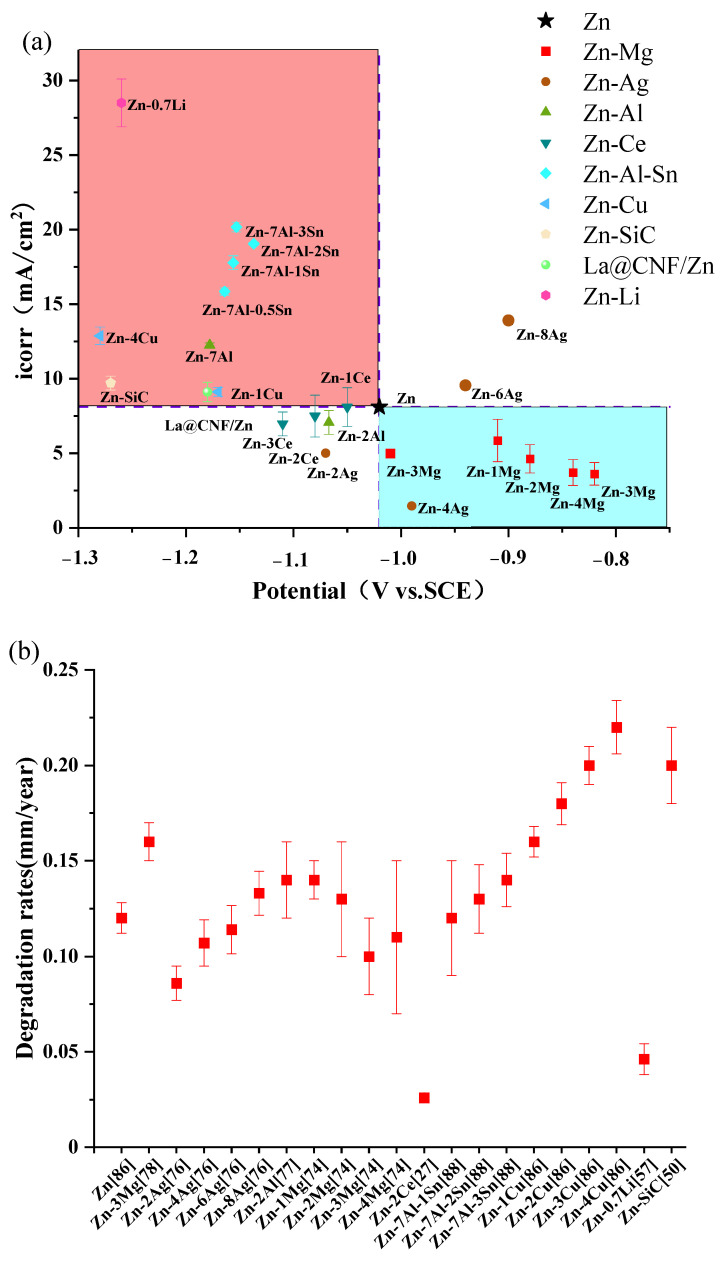
Corrosion properties and degradation rates of pure zinc and zinc alloys: (**a**) corrosion current density verses corrosion potential based on results of electrochemical test [27,50,51,57,74,76,77,78,86,87,89]; (**b**) In vitro degradation rates [27,50,57,74,76,77,78,86,88].

The results of electrochemical test and immersion test of LPBF Zn alloy in recent years are shown in the Figure 10 [27,50,51,57,74,76,77,78,86,87,89].

Zheng et al. [90] proposed a generalized reaction to characterize the degradation in the human body. The oxidation and cathode reactions are as follows when Zn is utilized:

(5) $$Zn\to {Zn}^{2+}+{2e}^{-}$$

(6) $$O_{2}+2H_{2}O+4e^{-}\to 4OH^{-}$$

Similar to Fe, Zn(OH)_2_ and ZnO can form corrosion products on the surface of Zn alloys through the following reactions:

(7) $${Zn}^{2+}+2{OH}^{-}\to Zn{\left(OH\right)}_{2}$$

(8) $$Zn{\left(OH\right)}_{2}\to ZnO+H_{2}O$$

The deterioration effect will occur in the process of Mg corrosion [34], whereas Zn does not liberate gas in this series of reactions. In addition, it has been documented that OH^-^ produced during the redox reaction might enhance the formation of calcium phosphates (CaP) precipitate, thereby improving the biological activity of Zn alloys [101,102,103].

In addition to the common Zn(OH)_2_ and ZnO products, Zn^2+^ react with various ions in the electrolyte to produce different corrosion products [98,104,105]. Corrosion products can sometimes act as corrosion barriers, reduces the rate of corrosion of metals. For example, the presence of an insoluble passivation film, like ZnHPO_4_, serves as a protective barrier for the underlying Zn metal and slows down the process of degradation [106]. The hydrozincite and simonkolleite have the ability to protective barrier that hinders the further degradation of the biodegradable Zn. Similar conclusions can be found in other literature [74,107,108].

### 4.3. Assessment of Biocompatibility

Biocompatibility refers to the characteristics of a substance that allow it to peacefully cohabit with living tissue, without inflicting significant injury or detrimental alterations to the living host. According to certain researchers, biocompatibility should encompass the ability of a material to effectively carry out its intended therapeutic function without inducing any negative side effects to the living host [109].

Currently, the research of LPBF Zn alloy mainly focuses on in vitro biocompatibility. Cytotoxicity tests (which assess the ability of a substance to damage cells) and blood compatibility tests (which evaluate the interaction between a substance and blood) are the most commonly used tests to determine in vitro biocompatibility. Human (e.g., aortic cells, osteoblasts, endothelial cells) and mouse (e.g., fibroblasts, osteoblasts) cells are used in cytotoxicity testing for Zn. The accurate selection of the appropriate cell line is essential in testing, as the results provide a precise comprehension of the implant’s practicality [110]. For instance, Cheng et al. [111] proposed that the cytocompatibility of Zn with human endothelial cells indicates the metal’s suitability for cardiovascular-related applications.

The following are common cytotoxicity tests: (1) extraction tests (e.g., MTT assay) to evaluate the toxicity of soluble substances in implants; (2) direct tests to evaluate the viability of cells growing in contact with the implant [112]. It has been mentioned in the literature that cytotoxicity is deemed acceptable if the cell viability exceeds 75%. For a period of 5 days, the researchers cultured MG-63 cells with 10% concentrations of LPBF Zn-3Cu, Zn-3Mg, Zn-3Nd, Zn-2Ce, and Zn-2Ag extracts. These MG-63 cells exhibited the characteristics of pseudopodium-rich, secreting extracellular matrix, with a cell viability exceeding 75% as the number of days increased. These findings suggest that the LPBF Zn alloy exhibits favorable cytocompatibility. The common in vitro biocompatibility studies of LPBF Zn are all about cytotoxicity tests, but the test methodologies employed by researchers are distinct. This may be associated with the addition of alloying elements, if known antibacterial elements (e.g., Cu, Ag) are employed, antibacterial tests are typically required. For instance, Zhao et al. [96] investigated the antibacterial properties of LPBF Zn alloy on staphylococcus aureus and colon bacillus. In comparison to pure Zn and Zn-Mg alloys, they discovered that Zn-Mg-Cu alloys showed superior resistance to pathogens. In addition, Blood compatibility testing, which involves the exposure of human blood to the implant, can obtain valuable information such as hemolysis (e.g., red blood cell destruction rate), platelet morphology, platelet adhesion, and dynamic blood clotting.

## 5. Biodegradable Zinc Alloys for Stent and Bone Implant Applications

The application of biodegradable Zn alloys in biomedicine is a relatively recent development. As early as 2007, Wang et al. [113] addressed the potential of Zn as a biodegradable implant in their research. The initial study of biodegradable Zn alloys was published by Vojtech et al. [100] in 2011, which initiated the formal research on the use of this metal in biodegradable implants.

The fabrication of stent typically commences with hollow microtubules, but there are different techniques for further fabrication of hollow microtubules. For instance, Hibel et al. [114] and Yang et al. [115] employed laser cutting, whereas Wang et al. [116] utilized a combination of laser etching and electrochemical refining to fabricate the stent. Because researchers are concerned about whether the process of turning hollow microtubules into stents will have adverse effects on mechanical properties and microstructure. Mostaed et al. [60] confirmed that the process of laser cutting does not result in significant changes in the microstructure and properties. This implies that the laser cutting technology is a feasible method for the manufacturing of Zn stents.

In the future, AM technology is expected to be the preferred method for stent fabrication, particularly in the production of customized implants for specific patients. Nevertheless, the current challenge is to enhance the forming quality and mechanical properties of the LPBF Zn stent, and secondly improve its biodegradation performance and biocompatibility. It has been reported that LPBF was employed to print Zn stent, and post-processing was implemented to enhance the surface roughness. For example, Wen et al. [25] engaged LPBF technology to fabricate pure Zn cardiovascular stent and implanted sandblasting treatment, as illustrated in Figure 9a [25,82,117,118,119,120]. It is evident that sandblasting is a challenging method for achieving an ideal surface roughness. However, it is feasible to address this porous structure using suitable polishing techniques, including chemical and electrochemical immersion polishing.

**Figure 11:**
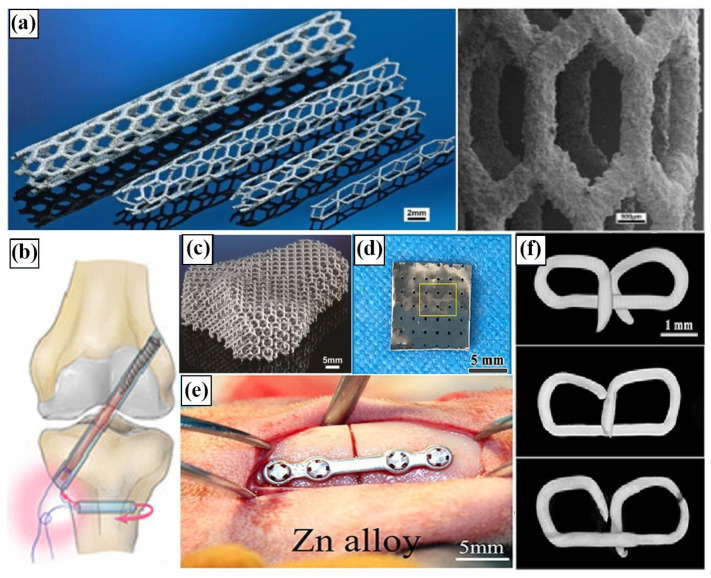
Stents and orthopedic implants based on Zn and its alloys: (**a**) SLM produced cardiovascular stents [25]; (**b**) anterior cruciate ligament reconstruction interface screw [117]; (**c**) part of the greater trochanter bone was prepared by SLM [82]; (**d**) skull defect repair scaffold [118]; (**e**) mandibular bone nail and bone plate [119]; (**f**) gastrointestinal anastomosis alloy nail [120]. (Reproduced with permission from Refs. [25,82,117,118,119,120], Elsevier).

The initial reports on the properties of Zn in vivo did not involve actual scaffold design, but rather simple shapes (e.g., wire) [9,121,122]. Liu et al. [36] reported tests of Zn mini tubes, which are the precursor shape of the scaffold, but no in vivo trials were conducted. In 2017, Yang et al. [115] published the first report on the performance of Zn scaffolds tested in vivo. The scaffolds were inserted into the abdominal aorta of rabbits and were subjected to a one-year period of testing. The recommendation of Zn as a biodegradable metal for future scaffold fabrication is a critical conclusion of this work. In a subsequent investigation, Zhou et al. [92] utilized femtosecond laser cutting to fabricate degradable Zn-0.8Cu coronary artery scaffolds, which were then implanted into the coronary arteries of pigs for 24 months. The scaffold was found to be intact during computerized tomography conducted at 6 months prior to and 9 months following implantation. Zn alloys stents and various orthopedic implants are shown in Figure 11 [25,82,117,118,119,120]. 

## 6. Results and Discussion

In order to realize the practical application of LPBF Zn implant materials, the requirements of mechanical properties, biodegradability and biocompatibility must be met. This section compares the data of literature, combined with the influence of process parameters and microstructure on the performance to find the appropriate method. Based on the existing results, the future research direction will be predicted.

### 6.1. Microstructure

The grain morphology is affected by SR and TR, as previously stated. The surface of LPBF alloys is typically composed to large columnar grains, which results in poor strength and toughness [26,123]. In order to achieve the optimal microstructure, numerous studies have adjusted a variety of factors (e.g., powder, process parameters, building direction) both prior to and during LPBF process. Nevertheless, the microstructure of the Zn alloy after printing can be modified once more via heating and cooling steps, which are known as heat treatment process. Grain refinement can be facilitated by the use of appropriate heat treatment procedure, which can promote grain boundary migration and inhibit grain growth. For instance, Cui et al. [124] performed a specific heat treatment on LPBF Zn to obtain an ideal grain size and phase composition. It is important to note that the mechanical properties of the parts can be significantly enhanced by the heat treatment procedure, as it eliminates the residual stress issue in the metal. Because in the LPBF process, rapid heating and cooling cycles may cause residual stress and thermal deformation of parts [125,126]. We believe that post-processing (e.g., heat treatment) is very helpful to the study of the properties of LPBF Zn alloy.

### 6.2. Mechnical Properties

The primary reason for the increase in the tensile strength of the Zn alloy is the presence of a second phase and the refinement of the grain. The second phase precipitates with small grain size can hinder the movement of dislocation without causing large stress concentration during deformation, thus enhancing the ability of precipitation strengthening and plastic deformation adaptation [127]. However, the second phase is a double-edged sword, which may reduce the elongation while increasing the tensile strength of the alloy. For instance, the tensile ductility of the Zn-3Mg alloy is diminished by the hard and brittle second phase, Mg_2_Zn_11_. Consistent results have been found in other studies [128]. The reason for the pool tensile ductility of the Zn-3Mg alloy is also its close-packed hexagonal crystal structure, which can operate at room temperature with a limited number of slip systems [129]. In addition to forming Mg_2_Zn_11_ brittle phase with Mg, Zn also forms brittle intermetallic compounds when alloyed with Ca, Sr, Al, Cu, Li and Ag [34]. As shown in the Table 4 [25,26,27,30,46,50,51,57,62,74,75,76,77,78,80,86,87,88,89,130,131,132], under normal circumstances, the yield strength and tensile strength of Zn samples will be significantly enhanced by increasing the alloy composition.

It is not difficult to comprehend that the mechanical properties of metals will be influence by the difference in particle size and second phase. However, it is crucial to know the mechanism by these properties are altered. The variation trend of mechanical properties of LPBF Zn can be predicted once the mechanical properties change law is mastered. Dislocation strengthening, solid solution strengthening, and grain boundary strengthening are the fundamental mechanisms that determine mechanical characteristics [41,133]. These fundamental mechanisms can provide a comprehensive explanation of the composition of strength, such as the Equation (9) for yield strength [134]:

(9) бy=бss+бg+бs+бd

where б_y_ is the yield strength, while б_ss_, б_g_, б_s_, and б_d_ are the contributions of solid solution strengthening, grain refinement strengthening, second phase strengthening, and dislocation strengthening respectively. The degree of the solid solution strengthening depends on the content of the alloying element dissolved in Zn. The maximum solubility of Mn in the Zn lattice is 0.8%. However, by the use of MA and LPBF process, a supersaturated solid solution Zn-Mn alloy has been created where approximately 3.6% of Mn is evenly distributed throughout the Zn lattice. The supersaturated solid solution Zn-Mn alloy’s compressive yield strength of 178 MPa is in line with the 110–180 MPa compressive yield strength of human bone [29]. Grain refinement strengthening can be expressed by Hall-Petch equation [135,136,137]:

(10) $${б}_{g}=k_{d}d^{-\frac{1}{2}}$$

where k_d_ is the strengthening coefficient (110 MPa μm^−1/2^) [138], d is the average grain size of the Zn alloy. Under specific circumstances, the yield strength of metal increases as the particle size decreases, as indicated by the equation.

The Orowan dislocation bypassing or shearing mechanism is the primary factor controlling the second phase strengthening. The second phase are skipped or sheared by moving dislocations under stress, which can be expressed as:

(11) $${б}_{s}=\frac{M\mu b}{L}$$

where μ is the shear modulus (39.5 GPa) [139], b is the Burgers vector (0.266 nm) [140], and L is the average size of precipitated phase particles, M is the Taylor factor for the basal texture of the Zn alloy.

The dislocation density and arrangement determine the extent of the internal stress generated by the rapid solidification of LPBF. The Bailey-Hirsh relation can be used to represent the strengthening of dislocations:

(12) $${б}_{d}=\alpha Mb\mu \sqrt{\rho }$$

where α is the dislocation interaction constant (0.2) [141], ρ is the dislocation density according to the EBSD analysis [142]. Although there is a certain gap between theoretical data and the actual value, this is an undeniable fact. However, the Equation (9) can provide researchers with numerous benefits, such as allowing researchers to find the cause of insufficient strength in time. In addition, the reliability of the Equation (9) has been confirmed by researchers, such as Zheng et al. [30]. The calculated theoretical data (597 MPa) is close to the compressive yield strength value of LPBF Zn-3Mg alloy measured by experiment (601 MPa). In conclusion, the ideal mechanical strength can be obtained by precisely adjusting the microstructure.

The structural design is a significant factor that influences the mechanical properties of porous scaffolds, and the mechanical properties just discussed are primarily focused on bulk Zn alloy. Porous scaffolds can be composed of crystal cells, and the performance of scaffolds varies with the structure of crystal cells. Lietaert et al. compared the mechanical properties of unit cells with five different structures (diamond, dodecahedron, octet truss, face centered cubic and 3D Kagome). The scaffolds with Kagome unit cells exhibited the highest strength, while those with oct truss unit cells exhibited the lowest strength. Researchers have combined unit cells of varying structures to create graded porous scaffolds, in addition to the porous scaffold composed of a single cell. For instance, the graded LPBF Zn-3Mg scaffold exhibits a compressive yield strength (170.75 MPa) and elastic modulus (20.3 GPa) that are significantly greater than the values reported in the literature for biodegradable porous Zn alloys [57,89,123,143]. Its compressive is comparable to that of human cortical bone [144]. To implement the use of Zn alloy implants, we can begin by examining the performance of unit cells with different structures and subsequently develop a functional scaffold with enhanced mechanical characteristics.

### 6.3. Biodegradation Performance

Table 4 [25,26,27,30,46,50,51,57,62,74,75,76,77,78,80,86,87,88,89,130,131,132] demonstrates the degradation rate in vitro, revealing that the corrosion performance of zinc alloy is influenced by the type and content of alloying elements. The inclusion of Mg decreased the corrosion rate, while the addition of Cu, Al, Sn, and Ag heightened the corrosion rate. Moreover, the corrosion rate of Zn alloy exhibits a pattern of increase and decrease with the increase of alloying element content. The influence of the kind and content of alloying elements on corrosion behavior appears to lack a definitive pattern.

The corrosion resistance of zinc alloy is also influenced by the distribution of the second phase and the size of the grains [145,146]. The influence of grain size on corrosion performance was documented by Gollapudi et al. [147]:

(13) $$i_{corr}=A+B{\left(d\right)}^{-\frac{1}{2}}\cdot exp\left(-\frac{9}{8}\right){S_{n}}^{2}$$

where i_corr_ is the corrosion current while A and B are two constants dependent on the material composition or impurity level, d is the average grain size, and S_n_^2^ is the grain size distribution. The Equation (13) demonstrates that a reduction in grain size will result in an elevation of the corrosion rate. When the alloying element content increases, the grain size tends to decrease first and then increase, which explains the reason why the Zn-Ag alloy rises first and then decreases. In addition, the corrosion rate of biodegradable metals can be influenced by other parameters, including grain orientation [148], residual stress [147], and specific surface area. Residual stress alters the thermodynamic state of the metal, hence influencing the rate of corrosion. Typically, the rate of corrosion rises as the residual stress increases [149]. As previously stated, heat treatment has the potential to decrease residual stress. However, it is still necessary to evaluate whether this would result in an increased corrosion rate. In terms of the corrosion rate of porous metal scaffolds, the specific surface area may be one of the most critical factors. The weight loss rate of LPBF Zn porous scaffolds is significantly higher than that of bulk Zn alloys under identical conditions, as a result of the surface’s expansion in contact with the corrosive medium.

Nevertheless, the validity of comparing the rates of degradation in controlled laboratory settings across many research and experimental situations is doubtful. Indeed, achieving a high level of consistency in the corrosion rates observed by polarization and immersion testing within a single study is challenging. This is because the corrosion behavior of the tested sample is influenced by various factors, including test settings, scanning rate, sample surface area, pH buffering technology, and flow conditions [150]. Several scholars have proposed that it is incorrect to directly compare the in vitro corrosion rates obtained from different methods [32].Thus, one of the pressing issues that has to be addressed is the establishment of a criteria for the biodegradability of bioabsorbable metals.

### 6.4. Biocompatibility

In general, the cell viability increases as the concentration of the extract. Further examination of more papers also confirms this occurrence. Tong et al. [151], Kubasek et al. [152]. Murni et al. [153], and Wang et al. [154] found that Zn extracts at a concentration of 100% exhibited significant cytotoxicity, but non-cytotoxicity was detected in extracts with lower concentrations. Furthermore, Table 5 [27,30,46,50,51,57,62,74,77,86,87,88,89,96,124] reveals that Zn exhibits excellent biocompatibility, even in extracts with a concentration of 100%. These disparities prompt the inquiry regarding the optimal concentration of extracts to employ in cell viability assays. According to Wang et al. [155], it is recommended to dilute extracts 6–10 times when assessing degradable metals. We consider this to be a justifiable statement as the test outcomes of the 100% extract have the potential to influence the researchers’ evaluation of the metal’s biocompatibility. Initiating research with low-concentration extracts as the primary focus can effectively reduce both the financial and temporal resources required by researchers. As shown in the Table 5 [27,30,46,50,51,57,62,74,77,86,87,88,89,96,124], it is evident that the cell viability of zinc alloy is closely correlated with the concentration of the extract.

Zn alloy will release Zn^2+^ during the corrosion process, and the influence of Zn^2+^ concentration on the in vitro cytocompatibility has been widely discussed. It is common sense that Zn^2+^ plays an important role in many basic cellular processes. [34,156,157,158]. The literature also indicates that Zn alloy implants can facilitate the proliferation and attachment of human mesenchymal stem cells (hMSC) [159,160]. Therefore, Yang et al. [161] assert that releasing a higher concentration of Zn^2+^ can promote bone healing. Nevertheless, several articles have proposed that excessive amounts of Zn^2+^ can potentially result in cytotoxicity and DNA damage to human osteoblast [153,162,163]. Hence, Zhu et al. [83] believe that high concentration of Zn^2+^ release may have harmful effects on cells, thereby leading to bone resorption in vivo. Based on their research, Ma et al. [164] discovered that a lower concentration (<80 μM) of Zn^2+^ enhances cell adhesion, but a larger concentration (80–120 μM) of Zn^2+^ hinders cell proliferation. All in all, during the corrosion of Zn alloys, we believe that a stable corrosion rate should be maintained to avoid huge fluctuations in the concentration of Zn^2+^.

Currently, there is a dearth of studies on the in vivo biocompatibility of LPBF Zn alloy, despite the fact that various studies have demonstrated its excellent in vitro cytocompatibility. This difference is understandable because in vitro experiments do not involve a live host, so the test conditions are relatively simple, inexpensive, can be standardized, and are suitable for large-scale screening [165]. In vivo experiments are frequently costly, time-consuming, and intricate. Nevertheless, in vivo investigations are indispensable and are regarded as a more pertinent method of evaluating the biocompatibility of potential implant materials.

### 6.5. Critical Evaluation

In general, the grain size of LPBF pure Zn is relatively large, and the grain form is thin columnar. This implies that Zn exhibits limited yield strength and anisotropy. Anisotropy can result in the premature failure of implants during their useful life, as they are required to withstand forces in all directions. With the addition of alloying elements, the presence of solid solution and second phase will make the columnar grain be replaced by equiaxed grain and the grain size decreases. The yield strength of Zn alloy is increased and the anisotropy is weakened, while the elongation is related to the generated second phase. With the increase of the content of alloying elements, alloying elements will reach and exceed the liquid to form a precipitated phase. This precipitated phase tends to increase the grain size and accelerate the rate of metal corrosion. After some researchers found this, Supersaturated solid solution was printed by LPBF from alloy powder prepared by MA [29]. When the mechanical properties of Zn gradually meet the requirements of bone implants, it is urgent to improve its degradation performance and biocompatibility. As a bone implant material, it is necessary to control the stability of the corrosion rate, provide the necessary Zn^2+^ to promote bone healing, and avoid the high concentration of Zn^2+^ to damage tissues and cells. At present, the primary objective of LPBF Zn is to conduct in vitro biocompatibility experiments. According to their experimental results, Zn has good cellular compatibility, and it is found that the cell viability increases with the decrease of extract concentration and the extension of culture period. Recently, in vivo compatibility test also has a good trend. The research status of in vivo compatibility experiments with LPBF porous scaffolds is as follows. Cui et al. [124], Qin et al. [89], and Zhao et al. [96]. implanted LPBF Zn alloy scaffolds into rabbit femur for 12 weeks. The results indicated that bone tissue regeneration was satisfactory and the implant structure remained intact. These studies indicate that LPBF Zn alloy scaffolds have a promising future as implant materials due to their favorable osseointegration and osteogenic properties.

In the review of biodegradable metals, the research direction and object of this paper is highly specific. This review allows readers to rapidly comprehend the current research status of LPBF Zn, gain a comprehensive understanding of the mechanical properties, corrosion properties, and biocompatibility of Zn test methods, as well as the underlying mechanism, and identify a breakthrough in future research.

## 7. Conclusions and Future Directions

An analysis of contemporary literature was conducted to assess the powder characteristics, microstructure, mechanical properties, degradation behavior, and biocompatibility of zinc alloys via LPBF. According to the current research status and development trend at home and abroad, in the future LPBF zinc alloy research, the following breakthroughs need to be made:

1. Performance of LPBF cardiovascular stent

As early as 2018, Wen et al. [25] used LPBF technology to print pure Zn cardiovascular stents, but there was no literature to continue the research. Initially, identify the appropriate process window by examining the formation quality of the cardiovascular stent. Secondly, with the help of finite element software simulation, the expansion and compression behaviors of different structures of cardiovascular stents were compared to obtain the optimal structure. Subsequently, the mechanical, in vitro corrosion, and in vitro biocompatibility properties of Zn and Zn alloy cardiovascular stents were investigated via LPBF.

2. Effect of post-processing on LPBF Zn

Currently, there are a multitude of studies investigating the properties of Zn alloys in terms of alloying elements, manufacturing technology, and process parameters. Subsequent researchers can utilize their process window and alloying element content as a reference to save time and generate more valuable results. Heat treatment is known to reduce metal grain size and eliminate residual stress. The researchers were able to control the duration of heating and cooling to find out how it affected the microstructure and mechanical properties of bulk Zn. The formation quality of porous Zn scaffolds is not easily improved by sandblasting, so the effects of surface treatment (e.g., coating, chemical and electrochemical polishing) on in vitro corrosiveness and in vitro biocompatibility can be studied.

3. Establish test standards for biodegradable metals

As discussed in the article, different test parameters, alloy samples all affect the biocorrosion behaviors and biocompatibility of biodegradable metals. Consequently, it is imperative to develop a test standard that accurately reflects the clinical application of implants. Its existence enables the effective comparison of the results of various studies and the easy identification of gaps, thereby facilitating future research.

## Figures and Tables

**Table 1:** Composition and technologies for additive manufacturing of Zn alloy.

Alloying Composition (at%)	Types of Additive Manufacturing Technologies
Zn-3Mg	LPBF
Zn-3Nd	LPBF
Zn-5WE3	LPBF
Zn-6Ag	LPBF
Zn-3Cu	LPBF
Zn-2Al	LPBF
Zn-2Ce	LPBF
Zn-0.7Li	LPBF
Zn-15Mn	LPBF
Zn-3Mg-2Cu	LPBF
Zn-7Al-2Sn	LPBF
La@CNF/Zn	LPBF

**Table 2:** The process parameters for SLM of Zn alloy [25,26,46,52,57,62,73,74,75,76,77,78,79,80].

Raw Material	P (W)	V (mm × s^−1^)	Hs (μm)	Ds (μm)	E_v_ (J/mm^3^)	Relative (Density%)	Refs.
Pure Zn	80	400	80	30	83.3	99.9	[52]
Pure Zn	100	300	100	30	111.1	99.86	[73]
Pure Zn	120	1400	35	20	122.45	96.67	[78]
Pure Zn	50	700	70	30	34.01	99.83	[79]
Pure Zn	90	500	70	30	85.7	99.91	[25]
Pure Zn	80	800	55	45	40.4	99.5	[62]
Pure Zn	100	800	70	30	59.52	93.04	[80]
Pure Zn	80	700	70	30	54.42	99.5	[26]
Zn-3Mg	200	200	80	100	125	98.2	[74]
Zn-3Mg	120	1400	35	20	122.45	95.99	[78]
Zn-10Mg	70	600	70	30	55.56	99.56	[79]
Zn-2Al	120	300	70	50	114.28	98.3	[77]
Zn-3Nd	50	300	60	70	39.7	98.71	[46]
Zn-5WE43	70	500	70	30	66.7	99.75	[75]
Zn-6Ag	70	12	120	100	486.1	97.9	[76]
Zn-0.7Li	40	800	70	20	35.71	99.5	[57]

**Table 3:** Compression properties of porous zinc alloy scaffolds prepared by LPBF [28,30,57,75,89,95,96].

Material	CYS (MPa)	UCS (MPa)	Young’s Modulus (GPa)	Porosity (%)	Refs.
Zn	12.7	22.9	0.95	45	[75]
Zn-2WE43	50.9	60.5	1.91	45	
Zn-5WE43	66.2	73.2	2.48	45	
Zn-8WE43	50.9	50.9	2.54	45	
Zn-1Mg	\	40.9	1.17	67	[89]
Zn-2Mg	\	35.3	1.34	67	
Zn-5Mg	\	23.6 ± 0.4	1.02	67	
Zn-0.7Li	\	18.2	2.98	80	[57]
Zn-Mg-Cu	41.1	43.5	2.18	\	[96]
Zn-3Mg	170.7	\	20.3	58.6	[30]
Zn	16.1	28	0.62	67	[95]
Zn-3Mg	48.3	50.5	2.37	67	
Zn	\	7.3	0.17	80.5	[28]
Zn-1Mg	\	19.2	0.65	80.5	
Zn-2Mg	\	16.5	0.49	80.5	
Zn-5Mg	\	6.2	0.41	80.5	

**Table 4:** Reported mechanical properties and intro biodegradability of LPBF Zn and Zn alloys [25,26,27,30,46,50,51,57,62,74,75,76,77,78,80,86,87,88,89,130,131,132].

Composition	Yield Strength, MPa	Tensile Strength, MPa	Elongation, %	Physiological Test Solution	Polarization Test CR, mm/y	Immersion Test CR, mm/y	Refs.
Zn	\	97	\	SBF	0.13	0.19	[78]
Zn-3Mg	\	197	\	SBF	0.09	0.16	
Zn	110	133	7.6	Hank’s	0.078	0.042	[26]
Zn	\	\	\	SBF	0.12	0.081	[76]
Zn-2Ag	\	\	\	SBF	0.08	0.086	
Zn-4Ag	\	\	\	SBF	0.02	0.107	
Zn-6Ag	\	\	\	SBF	0.15	0.114	
Zn-8Ag	\	\	\	SBF	0.21	0.133	
Zn-2Al	142	192	11.7	SBF	0.14	0.14	[77]
Zn	43	61	1.7	SBF	\	0.18	[74]
Zn-1Mg	74	126	3.6	SBF	\	0.14	
Zn-2Mg	117	162	4.1	SBF	\	0.13	
Zn-3Mg	152	222	7.2	SBF	\	0.1	
Zn-4Mg	132	166	3.1	SBF	\	0.11	
Zn	80	104	5.1	SBF	\	0.033	[27]
Zn-1Ce	131	196	5.8	SBF	\	0.028	
Zn-2Ce	181	247	7.5	SBF	\	0.026	
Zn-3Ce	192	233	6.7	SBF	\	0.024	
Zn-7Al	\	\	\	SBF	0.236	0.09	[88]
Zn-7Al-0.5Sn	\	\	\	SBF	0.305	0.1	
Zn-7Al-1Sn	\	\	\	SBF	0.342	0.12	
Zn-7Al-2Sn	\	\	\	SBF	0.367	0.13	
Zn-7Al-3Sn	\	\	\	SBF	0.388	0.14	
Zn	122	138	8.13	\	\	\	[25]
Zn	\	86	10.6	SBF	0.11	0.12	[86]
Zn-1Cu	\	148	15.8	SBF	0.135	0.16	
Zn-2Cu	\	182	18.4	SBF	0.158	0.18	
Zn-3Cu	\	223	22.5	SBF	0.17	0.2	
Zn-4Cu	\	207	21.4	SBF	0.19	0.22	
Zn-SiC	\	\	\	SBF	0.189	0.2	[50]
Zn	69	107	6.5	SBF	0.062	\	[51]
La@CNF/Zn	158	243	9.3	SBF	0.062	\	
Zn	70	83	3	SBF	0.095	\	[87]
Zn-2Cu	81	93	1.1	SBF	1.291	\	
Zn-0.7Li	359	417	\	Hank’s	\	0.046	[57]
Zn	\	116	26.1	Hank’s	\	\	[89]
Zn-1Mg	\	381	4.2	Hank’s	\	\	
Zn-2Mg	\	287	0.6	Hank’s	\	\	
Zn-5Mg	\	62	0.5	Hank’s	\	\	
Zn	66	83	5.6	SBF	0.52	0.2	[30]
Zn-3Mg	\	175	1.6	SBF	0.48	0.15	
Zn	\	67	10.2	SBF	\	\	[46]
Zn-1Nd	\	96	8.7	SBF	\	\	
Zn-3Nd	\	120	6.7	SBF	\	\	
Zn-5Nd	\	107	4.3	SBF	\	\	
Zn	\	134	10.1	\	\	\	[75]
Zn-2WE43	\	298	1.8	\	\	\	
Zn-5WE43	\	335	1	\	\	\	
Zn-8WE43	\	154	0.9	\	\	\	
Zn	84	96	11.7	\	\	\	[80]
Zn	100	128	7.6	SBF	\	0.046	[62]
Zn	\	\	\	SBF	1.09	\	[130]
Zn-0.6Li-0.5Mg	199	345	0.8	Hank’s	\	0.15	[131]
Zn	110	128	12.1	\	\	\	[132]

**Table 5:** Summary of reported in vitro biocompatibility of biodegradable LPBF Zn and Zn alloys [27,30,46,50,51,57,62,74,77,86,87,88,89,96,124].

Specimen	Cell Viability			Hemocompatibility	Other Tests	Refs.
	Cell Line	Exposure Times, d	Results			
Zn-2Al	Human osteosarcoma cells (MG 63)	1, 4, 7	Alloy was cytotoxic at 100% extract; noncytotoxic at 50% dilutions	\	\	[77]
Zn-x Mg (x = 1, 2, 3, 4)	Human osteosarcoma cells (MG 63)	1, 3	Cells exhibited good viability in all the specimens in 100% extract, and better viability in 50% extract of Zn-3Mg	\	\	[74]
Zn-x Ce (x = 1, 2, 3)	Human osteosarcoma cells (MG 63)	1, 3, 7	Zn-2Ce did not adversely affect cell viability	Both Zn and Zn-2Ce had good blood compatibility	Zn-2Ce exhibited an enhanced antibacterial effect as compared with Zn	[27]
Zn-7Al-xSn (x = 0.5, 1, 2, 3)	Human osteosarcoma cells (MG 63)	1, 3, 5	Cells exhibited good viability in Zn-7Al-2Sn	\	\	[88]
Zn-x Cu (x = 1, 2, 3, 4)	Human osteosarcoma cells (MG 63)	1, 3, 5	Cells exhibited good viability in all the specimens in 100% and 50% extract, and better viability in 10% extract of Zn-3Cu	\	Zn-xCu alloys exhibited strong antibacterial activity	[86]
La@CNF/Zn	Rat bone marrow stromal stem cells (BMSCs)	3, 7	La@CNF/Zn was cytotoxic at 100% extract; non-cytotoxic at 50% dilution	\	La@CNF/Zn displayed excellent anti-tumour efficiency	[51]
Zn-SiC	Human osteosarcoma cells (MG 63)	1, 3	Cells exhibited excellent viability in Zn-SiC at all exposure time	\	\	[50]
Zn-2Cu	Murine osteoblast precursor cells (MC3T4-E1)	1, 3, 5	Zn-2Cu showed low toxicity at all exposure time	\	\	[87]
Zn-0.7Li	Murine osteoblast precursor cells (MC3T4-E1)	1	i. Cell morphology showed good cell viability in Zn-0.7Li ii. porous sample shows better biocompatibility than bulk samples	\	\	[57]
Zn-x Mg (x = 1, 2, 5)	Murine osteoblast precursor cells (MC3T4-E1)	1, 3, 5	i. Zn and Zn-1Mg showed toxicity at 100% extract after 1 day ii. Cells viability of both specimens improved at 3 and 5 days	\	\	[89]
Zn-3Mg	Murine osteoblast precursor cells (MC3T4-E1)	1, 3	i. Cell viability decreased with increasing extract concentration	\	Zn-3Mg could promote osteoblast differentiation	[30]
Zn-x Nd (x = 1, 3, 5)	Human osteosarcoma cells (MG 63)	1, 4, 7	i. Cells exhibited good viavility in all the specimens ii. Cell viability improved with the extension of the culture period	\	Zn-Nd alloy had good anti-inflammatory activity	[46]
Zn	Rat bone marrow stromal stem cells (BMSCs)	1, 4, 7	Cells exhibited good viability in Zn at 100% extract	\	Zn exhibited good osteogenic differentiation ability	[62]
Zn	Murine osteoblast precursor cells (MC3T4-E1)	1, 7, 14	Heat treatment improved cell viability	\	\	[124]
Zn-Mg-Cu	\	\	\	\	Zn-Mg-Cu exerted excellent antibacterial efficacy against E. coli and S. aureus	[96]

## Data Availability

Not applicable.
